# Linguistic identity as a modulator of gaze cueing of attention

**DOI:** 10.1038/s41598-023-37875-7

**Published:** 2023-07-04

**Authors:** Anna Lorenzoni, Giulia Calignano, Mario Dalmaso, Eduardo Navarrete

**Affiliations:** Dipartimento di Psicologia dello Sviluppo e della Socializzazione, Università degli Studi di Padova, 35131 Padua, Italy

**Keywords:** Human behaviour, Psychology and behaviour

## Abstract

Eye-gaze stimuli can elicit orienting of attention in an observer, a phenomenon known as gaze cueing of attention. Here, we explored whether gaze cueing can be shaped by the linguistic identity of the cueing face. In two experiments, participants were first familiarized with different faces together with auditory sentences. Half of the sentences were associated with the native language of the participants (Italian) and the other half with an unknown language (Albanian and Basque, in Experiments 1 and 2, respectively). In a second phase, participants performed a gaze-cueing task. In a third recognition phase, the auditory sentences were presented again, and participants were required to decide which face uttered each sentence. Results indicated that participants were more likely to confuse faces from the same language category than from the other language category. Results of the gaze-cueing task revealed a greater gaze-cueing effect for faces associated with the native vs. unknown language. Critically, this difference emerged only in Experiment 1, which may reflect differences in social status between the two language groups. Our findings revealed the impact of language as a social cue on the gaze-cueing effect, suggesting that social attention is sensitive to the language of our interlocutors.

## Introduction

Individuals tend to orient their own attentional resources towards the same spatial location indicated by others^1^. This phenomenon, known as social attention, is a central ability, as it allows individuals to create meaningful social relationships and efficiently share attention towards a specific object or event occurring in the environment (e.g.^2^). An increasing number of studies has shown that eye-gaze direction is an effective cue of social attention, which provides a clear and easily accessible source of information about where another individual is attending (see for instance^3–5^ for reviews). This ability is considered to play a crucial role in social cognition^6^.

A standard method to study gaze-mediated orienting of attention is through the so-called gaze-cueing task (e.g.^7,8^). In this task, participants are typically presented with a central face with a direct gaze, and then with a picture of the same face with an averted gaze. Then, a peripheral target appears, requiring a manual response. In the so-called congruent condition, the target appears in the same spatial location indicated by gaze, whereas, in the incongruent condition, the target appears elsewhere. The classical results show that, even though the gaze direction is not informative on the location of the upcoming target, participants are faster and more accurate on congruent trials than on incongruent trials. This finding is interpreted to reflect an attentional shift in the direction signalled by the gaze (e.g.^7,8^).

Early studies considered the gaze-cueing effect a reflexive phenomenon, as it occurs even when the observer is not motivated to shift attention towards the direction cued by the gaze^7^, such as when gaze direction is counter-informative (i.e., targets are more likely to appear on the opposite side as that indicated by the gaze). However, more recent studies demonstrated that several social variables can modulate the gaze-cueing effect (for a recent review, see^9^). Indeed, in everyday life, we constantly interact with or are exposed to different people who may come from the same vs*.* different social environments as ours and who share the same vs*.* different social characteristics as ourselves. Information about age, gender, and ethnicity is automatically and rapidly extracted when we look at the face of a person^10,11^. Critically, this information contributes to categorize individuals, and it allows us to organize, structure, and process stimuli (e.g., faces) of our environment in a rapid and efficient manner^12–14^. Such a process of categorizing individuals (or faces) is known as social categorization. As already mentioned, previous studies showed that social information extracted from a face stimulus can shape the gaze-cueing effect. For example, a larger gaze-cueing effect has been reported for familiar faces over unfamiliar faces (e.g.^15^), for trustworthy faces over untrustworthy faces (e.g.^16^), and for faces described as belonging to high-status individuals rather than low-status individuals (e.g.^17,18^). Furthermore, there is evidence that group membership can also shape the gaze-cueing effect. For instance, Pavan et al.^19^ employed a gaze-cueing task in which White and Black faces were presented to White Italian and Black African participants living in Italy. The results showed that White participants exhibited a reliable gaze-cueing effect only in response to White faces. On the contrary, Black participants showed a reliable gaze-cueing effect regardless of the ethnicity of the cueing face (see also^20,21^). Interestingly for our purposes here, the study by Pavan and colleagues suggests that group membership and social status moderate the gaze-cueing effect. Indeed, as the authors pointed out, a possible explanation for these results could be derived from differences in social status attributed to the two groups: in Italy, White individuals are a majority and likely belong to higher status groups, while Black individuals are a minority and belong to a lower status group, respectively (see also^22^). Together, all these findings seem to confirm the important role of social factors in shaping gaze-mediated orienting of attention.

Recently, researchers have started to pay attention to a new dimension that may affect social categorization, that is, the language used by our interlocutors. Analogously to what has been observed with other cues, such as race and gender^10,11^, recent research has shown that individuals categorize others according to the language (or accent) they speak. This categorization appears to emerge in the first years of life, as evidenced by the observation that 6-month-old infants prefer looking at speakers of their same native language than those who speak a different language^23^. Other studies reported that 11- and 19-month-old infants, when learning new information, look more frequently at members belonging to the same linguistic group than at people of a different linguistic group^24–26^. With adult participants, research has shown that the language associated with a specific face stimulus is used to implicitly categorize individuals^27–30^.

In addition to what described above, empirical investigations on the role of language as a cue for categorization in adults have focused on the logic underlying the memory confusion paradigm (MCP^31,32^). The MCP is a standard way to implicitly measure social categorization, while removing social desirability effects^32–34^. The logic of the paradigm is that if a particular feature—such as language—is a cue that triggers categorization, then people who share the same dimension should be more confused with each other during a memory task. That is, when trying to recall specific information, memories of people who share the same language are more likely to be confused with each other, even in the absence of conscious awareness that this is happening. In this sense, patterns of memory confusion reveal fundamental categorization processes. The paradigm is traditionally divided into three sessions: familiarization, distractor task, and recognition. In the familiarization session, participants are exposed to pairings of faces and statements. Participants are simply told to make impressions of each person as they make each statement. Then, the distractor task is designed to prevent participants from explicitly thinking about the speakers and statements they had just seen. Finally, in the recognition session, participants see all faces they had seen previously and are asked to try to remember which statement came from which speaker (i.e., “Who said what?”). Unbeknownst to participants, errors in the recognition phase reveal non-conscious categorization processes. For instance, if a participant categorizes speakers by their language during the initial familiarization session, then during the recognition session they will be more likely to misattribute the statement to someone else who also spoke the same language as the original speaker, as opposed to someone who spoke in a different language. Using this paradigm, recent evidence has shown that categorization based on language (or accent) is an implicit and automatic process^27–30,35,36^. In sum, we orient attention in response to the eye-gaze direction provided by a face; at the same time, we categorize our interlocutors based on the language they speak. The aim of the present paper was to investigate whether these two processes interact.

In the present study, our objective was to investigate the role of language in guiding social attention. In particular, we explored whether the gaze-cueing effect was modulated by the linguistic identity associated with facial stimuli. To this end, we employed a gaze-cueing paradigm (e.g.^7^) and manipulated the linguistic identity of the cueing faces through a preliminary familiarization phase. In doing so, we wanted to shed fresh light on the top-down mechanisms influencing social attentional by adding to this debate one of the critical abilities of humans, that is, language. To ensure that faces were categorized according to language, we implemented the memory confusion paradigm by adapting it to the context of the gaze-cueing paradigm.

Overall, faster manual responses were expected in congruent trials than in incongruent trials, thus confirming the presence of a reliable gaze-cueing effect (see also, e.g.^7,8^). Critically, if the gaze-cueing effect is modulated by the linguistic identity associated with facial stimuli, we expected an interaction between the gaze-cueing effect and the linguistic identity associated with the cueing face. In addition, if faces were categorized according to language, we expected to replicate previous findings and to observe more same-language errors than different-language errors; that is, when participants make an error attributing a statement to a speaker, they are expected to be more likely to choose a speaker of the same language (see also, e.g.^27–30^).

According to the literature on gaze cueing (e.g.^9^), we believe that two alternative interpretations can be advanced for the possible interaction. One interpretation relies on the in-group vs*.* out-group distinction. In this regard, the respective membership of the face can shape the gaze-cueing effect depending on whether it belongs to the same group (in-group) or not (out-group) than the participant (see, e.g.^37^). In the context of this study, Italian participants may classify as in-group those faces that were associated with Italian sentences and as out-group those faces associated with the foreign language (Albanian and Basque). According to this scenario, a larger gaze-cueing effect is expected with in-group face stimuli, as was in the case in the study by Liuzza et al.^37^. At the same time, differences in the gaze-cueing effect when comparing two different social groups could be ascribed to asymmetries in their social status. Participants have been shown to shift attention more strongly in response to the averted gaze of a face that was described as depicting a high-status individual^17–19,22^. Thus, the status of the faces in our experiment can depend on the social status attributed to the two foreign languages by our Italian participants. In Italy, Albanian individuals represent a minority group and are often perceived as lower in social status than Italian individuals^38^. Basque, by contrast, is a language spoken mostly in Spain (see below) and the Italian population has probably little or no experience with the Basque speakers. More importantly, there is no a priori reason to expect Basque speakers to be considered as lower in status individuals compared to Italian speakers. To control for the role of status on the possible interaction between language and the gaze-cueing effect, our participants completed the MacArthur Scale of Subjective Social Status after the main gaze-cueing task.

## Experiment 1: Italian and Albanian languages

### Methods

#### Participants

Forty-eight Italian native speakers (24 females, mean age in years = 25.75, SD = 5.01) were recruited through the Prolific crowdsourcing platform^39^. The test was administered online and anonymously using Labvanced software^40^. All participants were required to give written informed consent. The inclusion criteria for all participants were: having Italian as a native language and having no knowledge of Albanian and Basque. In addition, participants had no reported cognitive, visual, or hearing impairments. The sample size was fixed to forty-eight participants according to the indication that, in a regression analysis (see the results section), increasing 5–10 observations per variable is likely to give at least an acceptable estimation of regression coefficients, standard errors, and confidence intervals^41–44^. In particular, the total number of observations in generalised linear mixed-effects models refers to both the number of participants and the number of observations nested within each participant per variable^45^. The research protocol was approved by the Ethics Committee of the Department of Developmental Psychology and Socialization, University of Padova (protocol number: 4505). All methods were performed in accordance with the relevant guidelines and regulations. All data are available under the following OSF repository: 10.17605/OSF.IO/ZCRVG.

#### Materials

Eight full-colour photos of adult Caucasian males with neutral expression were used as stimuli and were taken from the MR2 Face Database^46^. Photographs were divided into two sets. Within each set of four photos, photographs of faces were controlled for Attractiveness, Mood, Trustworthiness, Masculinity, and Age (all *p*s > 0.61). These images were then edited to remove the grey background and edit the direction of eye-gaze to create three versions of each face: straight, left, and right gaze. These stimuli were effective in eliciting a reliable gaze-cueing effect in previous studies (e.g.^47,48^).

In addition, twenty-four non-autobiographical sentences were created (e.g., “Frogs sing at night”). Half of them were recorded in Italian (native language) and the other half in Albanian (foreign language) using the software Audacity (v 2.0.3; https://www.audacityteam.org/). Sentences were different in the two languages, that is, they were not mere translations, to avoid any possible similitude between them (e.g., cognate words). Italian and Albanian audio tracks of the sentences were similar in length. Indeed, the recording durations of sentences in Italian [mean = 1.98 s, range = 1.79–2.42] and Albanian [mean = 2.22 s, range = 1.63–3.12] did not differ (t(22) = − 1.57, p = 0.13). To avoid any possible mismatch between face and voice, Italian and Albanian young adults, of similar age of the faces, were selected to record the sentences. In particular, four male native Italian speakers and four male native Albanian speakers recorded three sentences each. The final design consisted of photographs of faces accompanied by a voice speaking Italian or Albanian. Four lists were created to counterbalance face and language stimuli. The sentences and photographs together with the considered control variables can be consulted on the platform OSF.

#### Procedure

##### Gaze cueing and MCP tasks

**Figure 1:**
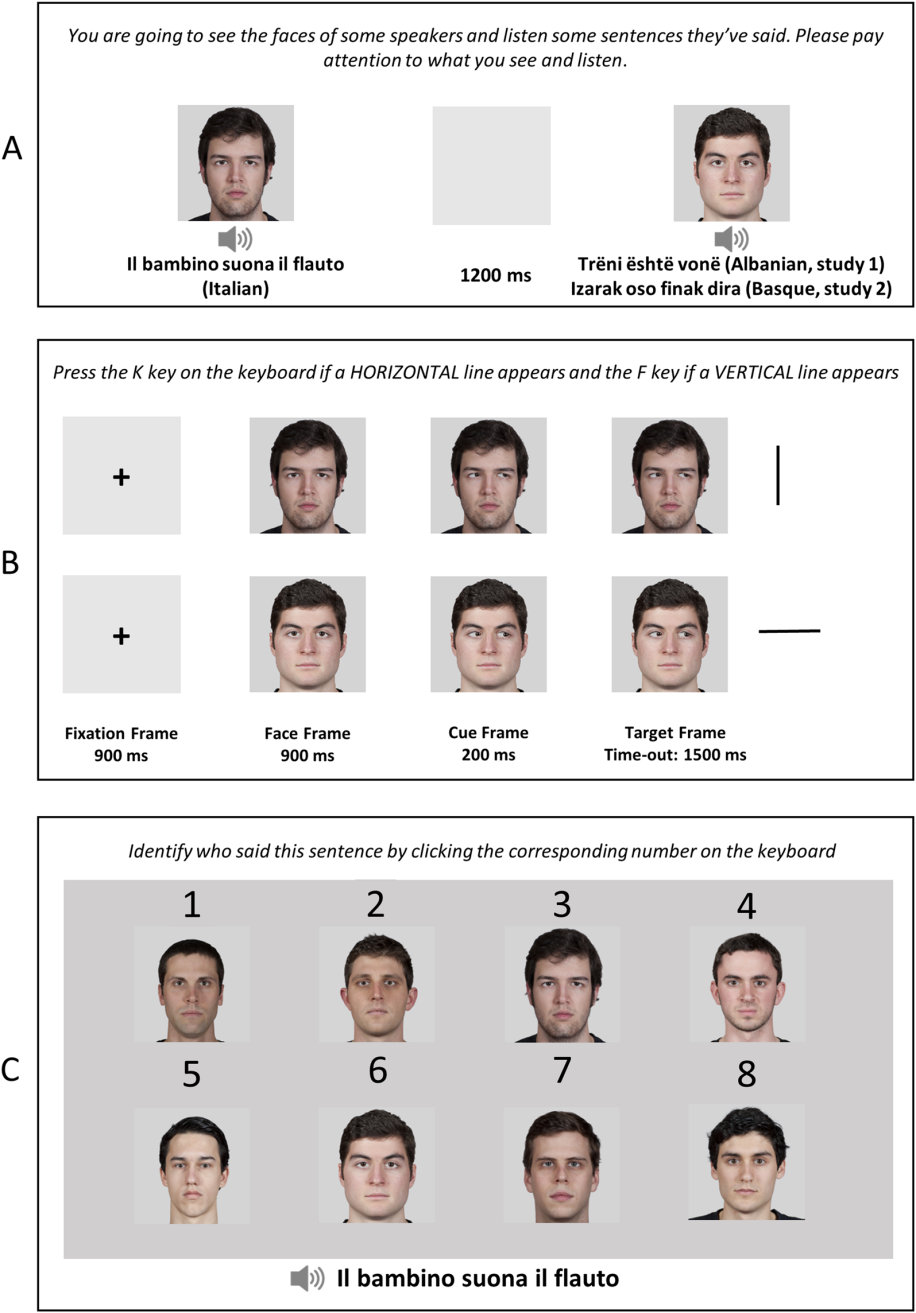
Illustration of stimuli (not drawn to scale) and sequence of events together with the given instruction to participants for the: (**A**) familiarization session; (**B**) gaze-cueing task and (**C**) recognition session. The faces shown in this figure are part of the MR2 Face Database^46^.

The experiment consisted of three sessions: the encoding session, the gaze-cueing task, and the recognition session (see also Fig. 1). Stimuli were presented on a grey background. At the beginning of the experiment, to avoid the interference of any sort of expectation and to preserve the implicit nature of the paradigms, the participant was only aware of the first session (i.e., the encoding session) and was informed that the study took approximately 25 min. Moreover, they were also informed about the nature of the two languages used in the experiment (i.e., Italian and Albanian). In the encoding phase, facial stimuli were presented on the screen one at a time along with the auditory presentation of the sentences. Participants only had to form impressions about the speakers as they watched and listened. The trial structure was the following: one photo and one audio were presented simultaneously. The photo of each speaker was displayed, centrally, for the duration of the statement, plus about two additional seconds thereafter, followed by a blank screen for 1200 ms. Each of the eight faces was presented three times during the encoding phase, for a total of 24 presentations. Additionally, each face was paired with a specific voice and associated with three different sentences spoken by that voice.

Upon completion of the encoding phase, participants were engaged in the gaze-cueing task in which the same eight faces were used as cueing faces. Each trial began with the presentation of a white fixation cross in the centre of the screen for 900 ms (fixation frame, Fig. 1), followed by a central face with direct gaze (face frame, 500 × 500 pixels). After 900 ms, the same face appeared with an averted gaze (cue frame). This photograph was obtained by moving the irises 0.25° to the right or to the left from the original central position using GIMP (v. 2.6). After 200 ms, a black line (horizontal or vertical, 0.82°) appeared 11° to the left or right of the centre of the screen in one of two possible locations: spatially congruent or incongruent with gaze direction. The target frame remained visible until a response was provided or for a maximum of 1500 ms, whichever came first. Participants were instructed that the direction of gaze was not informative in relation to the target location, and they were also asked to maintain fixation at the centre of the screen for the duration of the trial. The instructions emphasised both the response speed and accuracy. The participants responded using their right and left index fingers. Half of the participants were instructed to press the ‘K’ key on the keyboard if the target line was 'vertical' and the ‘F’ key if the target line was ‘horizontal’. The remaining participants responded using the opposite mapping. In case of a wrong or missed response, visual feedback (the words “ERROR” or “TOO SLOW”, respectively; Arial font) was provided at the centre of the screen for 500 ms. There were 64 trials for each condition defined by the spatial congruency between gaze direction and target location (congruent versus incongruent) and language (foreign versus native), for a total of 256 trials presented in random order. Literature on gaze cueing suggests that to observe an influence on gaze cueing of social variables that are arbitrarily associated with different facial identities, it can be necessary to reinforce that association through repeated exposure (see also^17,49^). Consequently, we decided to repeat twice the encoding phase (and, consequently, the gaze-cueing phase) to strengthen the association between language and facial stimuli. Thus, the gaze-cueing task was composed of two blocks (512 trials in total).

After the second gaze-cueing block, participants started the recognition phase, where all 8 photographs (225 × 225 pixels) were presented on the screen, numbered from 1 to 8. Face order was randomized among participants and trials. Then, the same 24 sentences from the encoding phase were presented again in auditory form. The participant decided which of the eight faces accompanied the sentence in the encoding phase by clicking on the keyboard the corresponding number. The eight faces remained on the screen until the participant’s response, after which a blank of 1000 ms was presented. The procedure continued until all 24 sentences in the encoding phase were presented. To summarize, the experiment consisted of five different phases: (1) an encoding session (block 1), (2) a gaze-cueing task (block 1), (3) a second encoding session (block 2, identical to phase 1), (4) a second gaze-cueing task (block 2, identical to phase 2) and (5) a recognition session.

##### MacArthur scale of subjective social status

To capture the possible role of social status associated with the languages used in our two Experiments, we asked our participants to rate the social status associated with the languages. Participants were contacted 15 days after the main experiment to fill out the MacArthur Scale of Subjective Social Status (MacArthur SSS Scale^50–53^), which provides a single item measure of the perceived social status of social linguistic groups. Participants were contacted 15 days after they had performed the main experiment to exclude any spurious effect of facial stimuli during the ratings. Participants in Experiment 1 and in Experiment 2 rated the social status of the three languages used in the main test of the two Experiments, that is, Italian, Albanian and Basque. The task was the following: Firstly, participants listened to four neutral sentences for each language, for a total of 12 sentences. These sentences were different from the experimental sentences used in the main experiments and were presented in a random order. Together with the sentences, the corresponding flag of the language was presented, that is, the flag of Italy, Albania, or the Basque Country. No faces were presented together with the sentences. Then, participants completed the MacArthur Subjective Social Status Scale for each language. Participants viewed a drawing of a ladder with 10 rungs together with the flag of the language and read that the ladder represented where people stand in society. More precisely, participants were provided with the following information: “At the top of the ladder are the people who are the best off, those who have the most money, most education, and best jobs. At the bottom are the people who are the worst off, those who have the least money, least education, worst jobs, or no job. By clicking on the number corresponding to the rung, indicate where you would place *[target language]* speakers on this scale.

#### Statistical analysis

##### Gaze-cueing task

We considered as experimental factors Gaze (Congruent vs. Incongruent), Language (Native vs. Foreign) and Block (First vs. Second). Block was added to consider possible learning effects in gaze cueing (see also^17,49^). Data from the gaze-cueing task were analysed using generalised mixed-effects models (GMMs^45^). GMMs are an extension of the general linear models (GLMs) that allow one to specify the distribution family. Since residuals are often positively skewed and heteroscedastic when dealing with nonnegative behavioural data (as, e.g., response time and accuracy), these models are preferred to the classical ANOVAs^54^. The GMMs approach allows modelling data for random and fixed effects. Moreover, those methods fit with multiple, crossed grouping factors and, possibly unbalanced data sets by stabilising the estimation of parameters^54,55^. To find the best approximation to the true model, we followed a model comparison approach with AIC (Akaike Information Criterion) and AIC weight as goodness-of-fit indexes. The AIC and AIC weight compare all the models at once and give information on a model's relative evidence (i.e., likelihood and parsimony), so that the model with the lowest differential AIC and the highest AIC weight is to be preferred^56^. We started from the simplest model with only random factors (participants and faces) and proceeded by adding predictors and, specifically, by weighting the effects of the main manipulations. To explore whether experimental manipulations statistically influenced response time, we visually inspected the model estimates of differences between conditions. We excluded anticipatory responses (< 100 ms) and included response times up to 1000 ms (1.72% of the trials were removed). Error trials (5.24%) were excluded from the response times (RTs) analysis and analysed separately. All data and analysis are openly available in the repository OSF link.

##### Recognition task

Following previous studies that have used this paradigm^27–30,35,36^, to test for the presence of a language effect, categorization was measured on a participant basis by calculating the difference in error rates between same-language errors and different-language errors. While there are only three possibilities to make same-language errors (because one of the faces is the correct answer), there are four possibilities to make a different-language error. To correct for this discrepancy, the number of different-language errors was multiplied by 0.75. Paired *t* test analyses were performed between same-language and different-language errors (see^57^ for validation of this method).

##### MacArthur scale of subjective social status

Linear mixed-effects regressions were performed on the ratings using the lme4 package^45^. In the mixed model, the factor “Language” was introduced as fixed effect, and Participant as random effect. We compared this model with a null model with only Participant as random effect.

### Results

#### RTs gaze-cueing task

**Figure 2:**
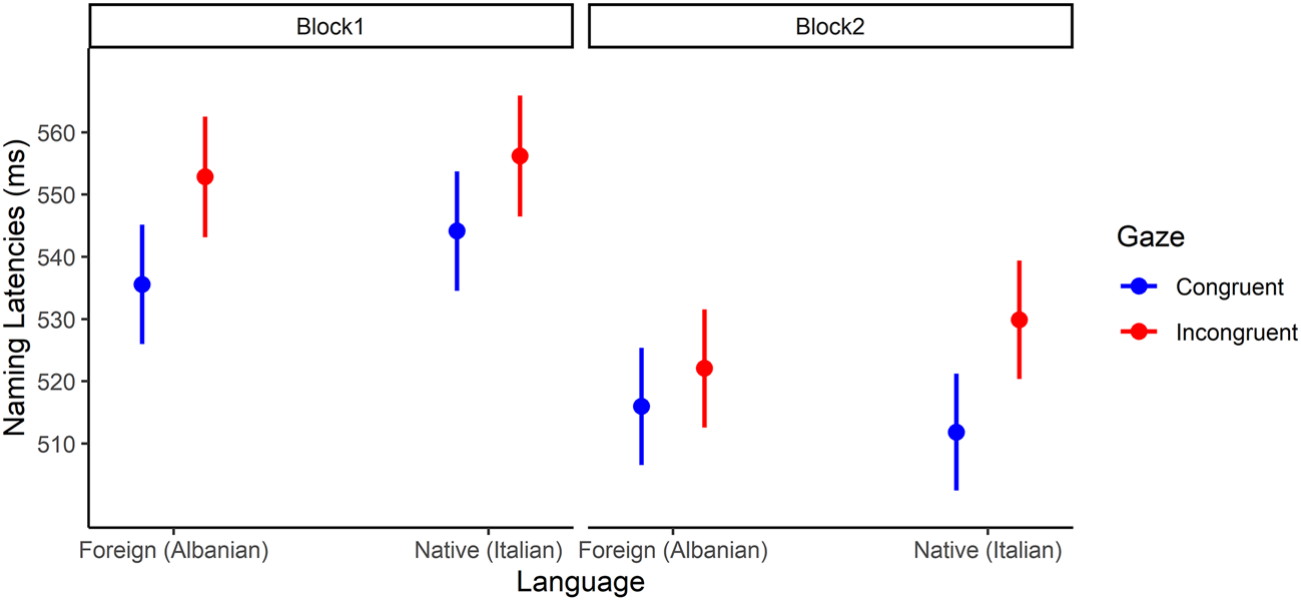
Marginal effects of interaction terms of the selected model for target detection time in milliseconds. The gaze-cueing effect was presented in the two blocks with native language faces, but it was absent in the second block with foreign faces.

The model of interest with the triple interaction between Gaze (Congruent vs. Incongruent), Language (Native vs. Foreign) and Block (1 vs. 2) was the most plausible predicting response times (b = − 17.17, SE = 8.09, t = − 2.12). In particular, the results from Block 1 showed a significant effect of cue-target congruency both for native (Incongruent as reference level, b = − 12.1, SE = 4.19, t = − 2.88) and foreign faces (b = − 17.3, SE = 4.15, t = − 4.17). However, for Block 2, this was true only when native faces (Incongruent as reference level, b = − 18, SE = 3.9, t = − 4.60) were presented (for foreign faces: b = − 6.1, SE = 3.92, t = − 1.56). Results from Block 2 revealed that participants shifted their attention in response to the averted gaze of native faces, but not in response to the averted gaze of foreign faces. See Fig. 2.

#### Accuracy gaze-cueing task

Analyses on accuracy were performed in order to exclude the presence of a speed-accuracy trade-off. The model with Gaze (Congruent vs. Incongruent), Language (Native vs. Foreign) and Block (1 vs. 2) was the most plausible predicting accuracy responses. Incongruent cues predicted a less accurate response compared to the Congruent cues (b = − 0.22, SE = 0.08, t = − 2.77). The results also show a significant effect of Block predicting a more accurate response for Block 2 than for Block 1 (b = 0.17, SE = 0.08, t = 2.20). No effect of Language was found (b = 0.05, SE = 0.08, t = 0.68).

#### Recognition task

**Figure 3:**
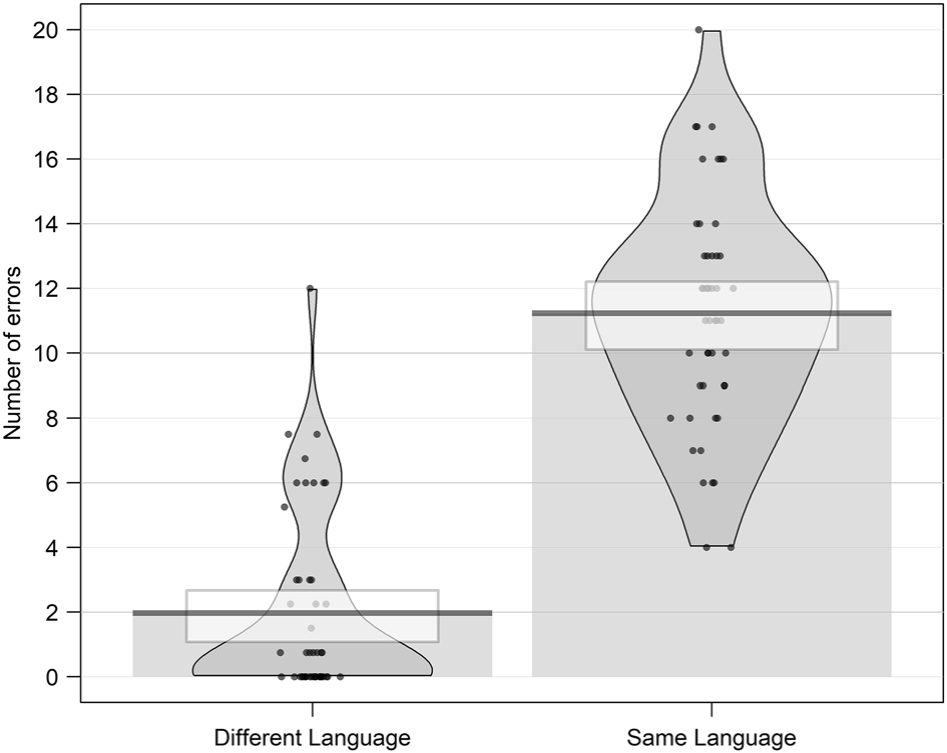
Mean of errors, committed during the recognition task, split by type of error for Experiment 1.

The paired *t* test showed that participants made significantly more same-language errors (11.23, SD = 3.58) than different-language errors (1.97, SD = 2.85, t(47) = 12.45, p < 0.001). See Fig. 3. In addition, results from linear regression on correct responses revealed that participants made significantly more correct answers with a native with respect to a foreign face (b = 2.27, SE = 0.52 t = 4.34).

#### MacArthur of subjective social status scale

Results showed that Albanian was judged the language with the lowest status (M = 5.21; SD = 1.72), compared to both Basque (M = 6.19; SD = 1.08; b = − 0.97, SE = 0.20, t = − 4.78) and Italian (M = 6.80; SD = 1.03; b = − 1.58 SE = 0.20, t = − 7.77) languages. Basque was judged with lower status than Italian (b = − 0.61, SE = 0.17, t = − 3.65).

### Discussion

Two main findings emerged from this experiment. First, the results of the recognition task revealed that participants categorized faces based on the language they were associated with, thus replicating recent findings on the role of language as a cue for social categorization^27–29^. Second, and more importantly here, the results from the gaze-cueing task revealed that the language associated with facial stimuli shaped the magnitude of the gaze-cueing effect. In particular, we obtained evidence showing that the gaze-cueing effect for facial stimuli associated with the foreign language was abolished. This is in line with our hypothesis based on in-group vs*.* out-group distinction (e.g.^37^) and, more generally, with the idea that people would be more inclined to prefer and prioritise own-language speakers than foreign language speakers^58–62^. The modulatory role of language on gaze cueing reported in Experiment 1 was detected only in the second block, namely, after that participants completed two learning phases aimed at associating a given language with a specific face identity. This seems to confirm that this association would require to be reiterated to fully emerge and be detectable at the attentional level, which is in line with some previous works on gaze cueing^17,49^. Finally, according to the results from the MacArthur Social scale*,* Albanian was judged with lower status scores compared to Italian (and Basque). This latter evidence suggests that our results can be also interpreted in terms of differences in social status (see also, e.g.^17,18^).

In the next Experiment, we wanted to extend and clarify the results observed in Experiment 1 with a different pair of languages: Italian (native language) and Basque (foreign language). The Basque language is spoken by individuals living in the Basque Autonomous Community and Navarra in north-eastern Spain and in some areas in south-western France. According to our original prediction, participants in Experiment 1 judged Albanian language as the lowest in social status. However, they also judged Basque language as slightly lower in status than Italian language, which was unexpected. We tested the robustness and reliability of these results by administering the *MacArthur Scale* also to the new pool of Italian participants of Experiment 2. In relation to the gaze-cueing task, if the results observed in Experiment 1 were driven by an own-language vs. foreign language distinction, that is, an in-group/out-group distinction, a similar modulation on the gaze-cueing effect should have emerged in Experiment 2 (i.e., an abolished gaze-cueing effect, in the second block, for the faces associated with the Basque language). Otherwise, if the results observed in Experiment 1 were driven by differences in social status between the two linguistic groups, a different scenario could emerge in Experiment 2, according to the results provided by the *MacArthur Scale*. If the status associated with the Basque language was perceived, again, as closer to the status of Italian as compared to the status of Albanian, then the gaze-cueing effect for the faces associated with Basque could be just reduced (or even be unaffected, if the difference in status was too small to detect an effect at the attentional level) in the second block, and not abolished as for the faces associated with the Albanian language (Experiment 1).

## Experiment 2: Italian and Basque languages

### Methods

#### Participants

The sample size was identical to that used for Experiment 1. Hence, a novel sample of forty-eight adults (24 females, mean age in years = 25.70, SD = 4.75) were also recruited and tested in this second experiment. Participants come from the same Italian pool of participants of Experiment 1 and were recruited through the Prolific crowdsourcing platform. The same inclusion criteria as in Experiment 1 were applied in Experiment 2. None of the participants involved in Experiment 1 took part in Experiment 2.

#### Materials

Everything was identical to Experiment 1, with the following exception: the sentences in Albanian language were replaced by other twelve sentences recorded by four male native Basque speakers, which therefore became the foreign language (e.g., “The cell phone fell to the floor”). Recording durations of sentences in Italian [mean = 1.98 s, range = 1.79–2.42] and Basque [mean = 2.01 s, range = 1.60–2.29] did not differ (t(22) = 0.37, p = 0.71). The final design consisted of photographs of faces accompanied by a voice speaking either Italian or Basque.

#### Procedure

Everything was identical to Experiment 1.

#### Statistical analysis

Everything was identical to Experiment 1. As for the gaze-cueing task, we excluded anticipatory responses (< 100 ms) and included response times up to 1000 ms (1.79% of the trials were removed). Error trials (6.17%) were removed and analysed separately.

### Results

#### RTs gaze-cueing task

**Figure 4:**
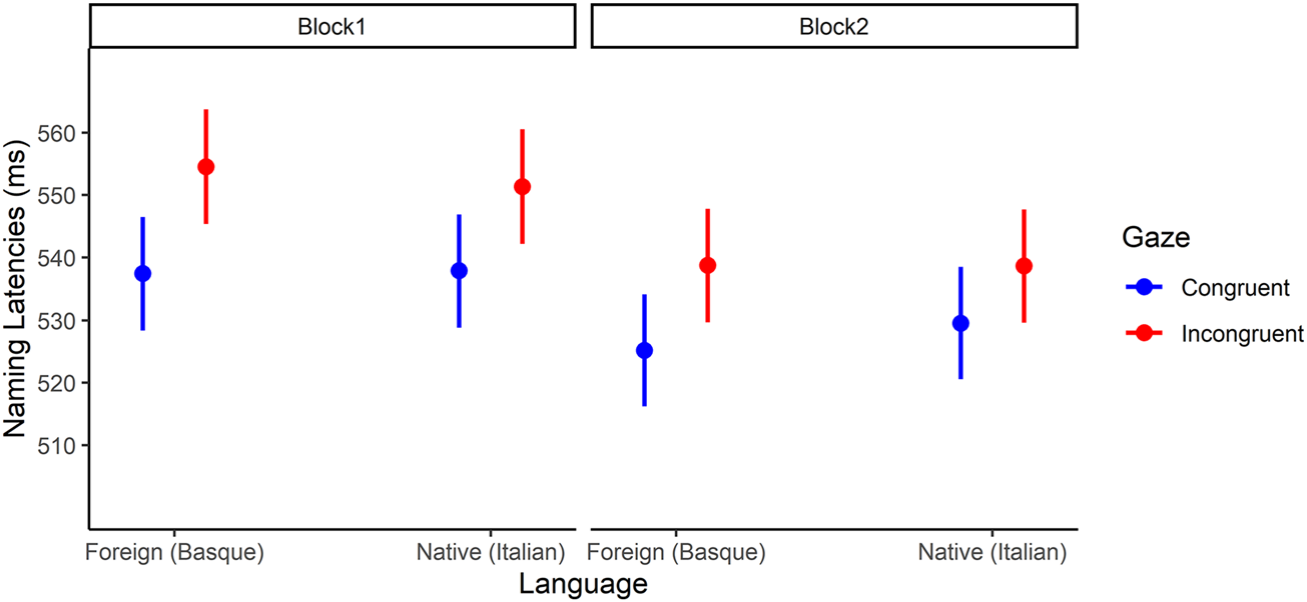
Marginal effects for target detection time in milliseconds. A similar gaze-cueing effect emerged in both blocks, and for the faces associated with the foreign and the native languages.

The model with Gaze (Congruent vs. Incongruent), Language (Native vs. Foreign) and Block (1 and 2) was the most plausible predicting response time. Incongruent cues predicted a slower response compared to the Congruent cues (b = − 13.26, SE = 2.02, t = − 6.57). The results also show a significant effect of Block predicting faster response time for block 1 than for block 2 (b = − 12.22, SE = 2.02, t = − 6.05). No effect of Language was found (b = 0.51, SE = 2.02, t = 0.25). Figure 4 shows the differential effect plot for each of the three factors.

#### Accuracy gaze-cueing task

Analyses on accuracy were performed in order to exclude the presence of a speed-accuracy trade-off. The model comparison revealed that the best model is the null model. Neither the gaze effect (b = − 0.09, SE = 0.07, t = − 1.24) nor the language effect (b = 0.03, SE = 0.075, t = 0.42) were significant.

#### Recognition task

**Figure 5:**
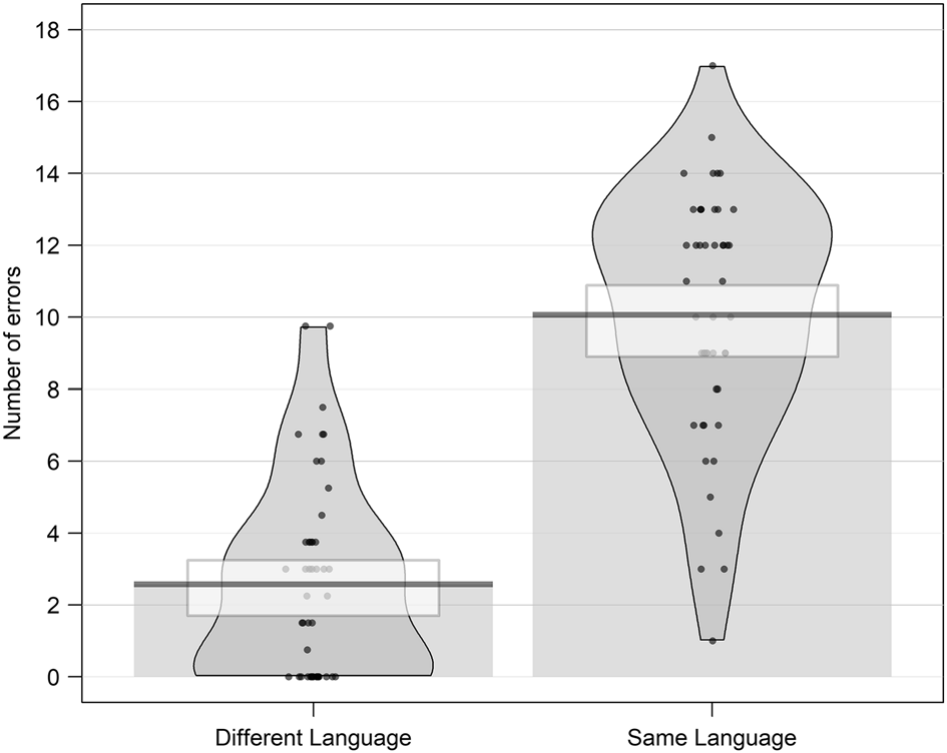
Mean of errors, committed during the recognition task, split by type of error for Experiment 2.

The paired *t* test showed that participants made significantly more same-language errors (10.06, SD = 2.73) than different-language errors (2.58, SD = 3.46, t(47) = 12.05, p < 0.001). See Fig. 5. In addition, results from linear regression on correct responses revealed that participants made significantly more correct answers with a native with respect to a foreign face (b = 1.37, SE = 0.60, t = 2.30).

#### MacArthur scale

Results showed that Albanian was judged the language with the lowest status (M = 5.53; SD = 1.63), compared to both Basque (M = 6.29; SD = 1.08; b = 0.75, SE = 0.25, t = 3.06) and Italian (M = 7.09; SD = 1.26; b = 1.55 SE = 0.24, t = 6.31) languages. Basque was judged with lower status than Italian (b = 0.80, SE = 0.21, t = 3.77).

### Discussion

The results of the recognition task revealed that participants categorized faces based on the language they were associated with, replicating Experiment 1. In addition, as in Experiment 1, participants judged Italian as the language with higher status, followed by Basque and then Albanian. However, gaze cueing of attention was not modulated by language identity, indicating that faces associated with Italian and Basque language had a similar effect at the attentional level. In the further section we discuss possible explanations for these results.

## General discussion

The purpose of the present study was to investigate the possible role of language in shaping social attention. In particular, we explored whether the gaze-cueing effect was modulated by the linguistic identity associated with facial stimuli. In two Experiments, we employed a gaze-cueing paradigm and manipulated the linguistic identity of the cueing faces through a preliminary familiarization phase in which participants listen to sentences of different languages and, at the same time, they also saw the faces of possible speakers. In the two Experiments, faces could be associated with the native language of the participants (i.e., Italian) or with a foreign and unknown language (i.e., Albanian in Experiment 1, and Basque in Experiment 2). Faces associated with the foreign language did not elicit a gaze-cueing effect, as compared to the faces associated with the native language, in Experiment 1 alone. In Experiment 2, in contrast, no differences in the gaze-cueing effect emerged between the two groups of faces. In addition, at the end of the gaze-cueing task, participants were instructed to identify which face was associated with each sentence using the memory confusion paradigm. Results from both Experiments revealed that participants implicitly categorized the faces based on the language they were associated with in the familiarization phase. Overall, these results confirmed and extended the knowledge on the role of linguistic identity in shaping both mnemonic and attentional mechanisms.

The novel result emerging from this work was that gaze cueing of attention was likely modulated by the linguistic identity in Experiment 1, suggesting that linguistic identity is a critical cue during social attention. To our knowledge, this is the first evidence that implicit linguistic categorization affects social attention. The interaction between linguistic identity and gaze cueing was absent in Experiment 2. That is, a plausible explanation for the different patterns found in the two Experiments may also be explained by the lower social status attributed to Albanian (Experiment 1) individuals compared to Basque individuals (Experiment 2). This difference appeared to be confirmed by the self-report measures we collected from our samples, showing that the social status of Albanians was perceived as lower than the social status of Basques and Italians (see also^38^). Although Basque was also perceived to be lower in social status compared to Italian, no interaction between language and gaze cueing emerged in Experiment 2. We argued that this could be due to the fact that the difference was not enough to modulate gaze cueing. In fact, when the two experiments were carried out, the difference in social status between Italian and Basque was half (0.71) than the difference between Italian and Albanian (1.57).

It is well-known that humans are particularly sensitive to social hierarchies (see, e.g.^63^), likely because high-status individuals are perceived to be considered as more relevant sources of information when compared with low-status individuals. According to this notion, it has been indicated that people tend to look at high-status individuals more often and for longer than at low-status individuals^64^, and also that gaze cueing of attention is magnified when elicited by faces associated with high status than low status^17,18,21^. Therefore, in the present context, it seems reasonable to assume that the different social status associated with Albanians and Basques individuals may have influenced the attentional response to eye-gaze stimuli provided by the groups of faces. Critically, eye-gaze stimuli in our Experiments affected participants differently based on the implicit categorization that they made during the familiarization task, given that all other conditions were identical in both experiments.

## Conclusion

To conclude, our results revealed the role of language in social attention. This agrees with previous studies showing top-down influences in social attentional^9^. Our research contributes to this debate by testing one of the most critical human abilities: language. Future research should address the robustness of this data pattern by further exploring the possible interplay between language, group affiliation, and social status. In particular, future studies will aim to investigate how the individual attentional cueing changes in the presence of faces considered outgroup with a specific social status. The results of the present study suggest that there are indeed context-specific influences on the gaze-cueing effect of faces belonging to different language groups, and that these influences are likely linked to hierarchical differences presented within the specific social context in which a language is spoken. Overall, exploring the possible role of linguistic identity in gaze cueing of attention is crucial to foster our understanding of interpersonal communication and social attention mechanisms.

## Data Availability

All data is available under the following OSF repository: 10.17605/OSF.IO/ZCRVG.
